# Draft Whole-Genome Sequences of Two Western European Borrelia miyamotoi Isolates

**DOI:** 10.1128/MRA.01314-19

**Published:** 2019-12-12

**Authors:** Konstantin V. Kuleshov, Dieuwertje Hoornstra, Hein Sprong, Alexander E. Platonov, Joppe W. Hovius

**Affiliations:** a Central Research Institute of Epidemiology, Moscow, Russia; b Federal State Budget Scientific Institution Federal Scientific Center VIEV, Moscow, Russia; c Amsterdam University Medical Centers, Amsterdam, The Netherlands; d Center for Infectious Disease Control, National Institute for Public Health and the Environment (RIVM), Bilthoven, The Netherlands; University of Maryland School of Medicine

## Abstract

We report the draft whole-genome sequences of two Borrelia miyamotoi strains isolated in The Netherlands. Using next-generation sequencing, we determined the complete sequence of the chromosomes and several plasmids. The two strains show a genotype typical of European strains, distinct from the genomes of strains from Asia or the United States.

## ANNOUNCEMENT

Molecular analysis of the emerging human hard tick-borne pathogen Borrelia miyamotoi (1) is cumbersome, since at present a limited set of isolates are available and have been sequenced (2, 3). Thus far, the genomes of 17 B. miyamotoi isolates have been published, including 12 from Russia, 1 from Japan, and 4 from North America. No whole-genome sequences (WGSs) of Western European isolates have been described.

We collected approximately 100 adult female Ixodes ricinus ticks in The Netherlands in 2018; the ticks were fully fed under laboratory conditions on rabbits or collected from domestic animals. The acquired egg masses were cultured in modified Kelly-Pettenkofer medium supplemented with 10% fetal calf serum (FCS) and selection antibiotics (4). We obtained two B. miyamotoi isolates, NL-IR-1 and NL-IR-2, from ticks collected in Amersfoort and Veldhoven, The Netherlands, respectively. Low passage numbers of the isolates were cultivated *in vitro* and washed in phosphate-buffered saline; the total DNA was then extracted using the Qiagen-tip 100 preparation kit. The DNA libraries were prepared by using a Nextera XT DNA library kit and sequenced through the MiSeq platform and 500-cycle v2 reagent kit (Illumina). *De novo* assembly was performed by using SPAdes v3.9.0 with default parameters for paired-end reads (5). Annotation of contigs was performed by using the NCBI Prokaryotic Genome Annotation Pipeline (PGAP) v4.9 (6).

The accession numbers, assembly metrics, and annotation features of the two sequenced Western European B. miyamotoi isolates are described in Table 1. Comparison analysis with the previously sequenced genome of CT13-2396 using Mummer v3, with the maxmatch option and minimal clusters of 100  nucleotides, resulted in contigs of both strains including the complete linear chromosomes of 903,844 bp (NL-IR-1) and 905,334 bp (NL-IR-2) and complete plasmids (lp72, lp6), in combination with the partially sequenced (left part) virulence plasmid lp41, containing the variable large protein (*vlp*) delta subfamily after the expression site (7). The remaining contigs likely represented fragments of other plasmid sequences, which were not checked for circularity. Through an InterProScan v5.23-62.0 search as described by Koetsveld et al. (8), we identified 39 coding DNA sequences (CDSs) and 8 pseudogenes of *vlp* genes and 16 CDSs of variable small protein (*vsp*) genes in the NL-IR-1 genome, whereas in the NL-IR-2 genome, we found 37 CDSs and 11 pseudogenes of *vlp* genes and 14 CDSs of *vsp* genes.

**TABLE 1:** Accession numbers, assembly metrics, and annotated features of the two sequenced Western European Borrelia miyamotoi isolates

Isolate name	GenBank accession no.	Avg sequencing depth (×)	No. of contigs	Total no. of reads	Genome assembly size (bp)	*N*_50_ value (bp)	G+C content (%)	No. of genes	No. of pseudogenes	No. of chromosomal genes (no. of CDSs)	No. of rRNAs	No. of tRNAs	No. of ncRNAs^*a*^
NL-IR-1	CP044783–CP044956	693	174	6,937,400	1,331,067	903,844	28.5	1,348	82	844 (807)	3	31	3
NL-IR-2	CP044625–CP044782	937	158	7,899,264	1,292,279	905,334	28.5	1,290	75	847 (810)	3	31	3

a ncRNAs, noncoding RNAs.

**FIG 1:**
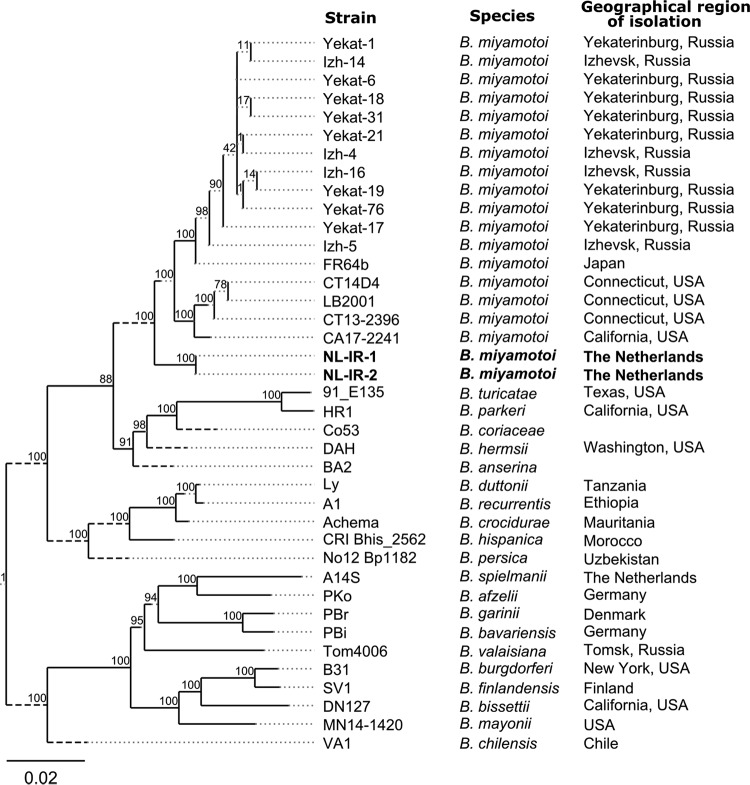
Concatenated core genes (*n* = 230) among 39 *Borrelia* genomes, including 19 Borrelia miyamotoi genomes, were generated through Roary v3.11.2 with the “-i 70” option (minimal identity percentage for BLASTP). A maximum likelihood (ML) phylogenetic tree was constructed using the RAxML software, employing a nucleotide substitution model with a gamma distribution of various positions (GTR + Γ) and the “-f a” option for rapid bootstrap analysis and to search for the best-scoring ML tree. The resulting tree was midpoint rooted (http://tree.bio.ed.ac.uk/software/figtree/). Cut long branches indicated by dashed lines. Scale bar indicates substitution rates.

Phylogenetic analysis based on the core gene alignment of the complete chromosome sequences of B. miyamotoi genomes of different origin in GenBank resulted in a separate phylogenetic lineage for the European B. miyamotoi strains (Fig. 1). Different numbers of single nucleotide polymorphisms (SNPs) (ranging from 16,000 to 18,000) in the core genes of the complete chromosome sequences were detected when comparing the Dutch strains to the Russian and Japanese (FR64b) or American (LB-2001, CT14D4, CT13-2396, and CA17-2241) strains. This significant genetic distance might indicate a distinct evolutionary pathway of the Western European B. miyamotoi population. Notably, the 3 phylogenetically different B. miyamotoi groups from Western Europe, Asia, and the northeastern coast of the United States are transmitted by different *Ixodes* ticks (*I. ricinus*, I. persulcatus, and I. scapularis, respectively) (9). Our findings provide a solid basis for future comparative genomics of B. miyamotoi isolates worldwide. Additional long-read sequence approaches are mandatory for completing the genomes and reconstructing the complex plasmid composition of these Western European strains.

### Data availability.

The raw sequencing reads of isolates NL-IR-1 and NL-IR-2 have been submitted to the SRA under accession numbers SRX6950008 and SRX6950009, respectively. The annotated sequences of the chromosomes and (in)complete plasmids are uploaded to GenBank/DDBJ/EMBL under accession numbers CP044783 through CP044956 and CP044625 through CP044782 for NL-IR-1 and NL-IR-2, respectively, with BioProject accession number PRJNA573610 and BioSample accession numbers SAMN12826994 and SAMN12826993.
